# Expression profiling of cervical cancers in Indian women at different stages to identify gene signatures during progression of the disease

**DOI:** 10.1002/cam4.152

**Published:** 2013-10-31

**Authors:** Asha Thomas, Umesh Mahantshetty, Sadhana Kannan, Kedar Deodhar, Shyam K Shrivastava, Chandan Kumar-Sinha, Rita Mulherkar

**Affiliations:** 1 Advanced Centre for Treatment, Research and Education in Cancer (ACTREC), Tata Memorial CentreNavi Mumbai, Maharashtra, India; 2 Department of Radiation Oncology, Tata Memorial Hospital, Tata Memorial CentreMumbai, Maharashtra, India; 3 Department of Pathology, Tata Memorial Hospital, Tata Memorial CentreMumbai, Maharashtra, India; 4 Department of Pathology, Michigan Center for Translational Pathology, University of MichiganAnn Arbor, Michigan

**Keywords:** Biomarker, cervical cancer, expression profiling, microarray, real-time PCR

## Abstract

Cervical cancer is the second most common cancer among women worldwide, with developing countries accounting for >80% of the disease burden. Although in the West, active screening has been instrumental in reducing the incidence of cervical cancer, disease management is hampered due to lack of biomarkers for disease progression and defined therapeutic targets. Here we carried out gene expression profiling of 29 cervical cancer tissues from Indian women, spanning International Federation of Gynaecology and Obstetrics (FIGO) stages of the disease from early lesion (IA and IIA) to progressive stages (IIB and IIIA–B), and identified distinct gene expression signatures. Overall, metabolic pathways, pathways in cancer and signaling pathways were found to be significantly upregulated, while focal adhesion, cytokine–cytokine receptor interaction and WNT signaling were downregulated. Additionally, we identified candidate biomarkers of disease progression such as SPP1, proliferating cell nuclear antigen (PCNA), STK17A, and DUSP1 among others that were validated by quantitative real-time polymerase chain reaction (qRT-PCR) in the samples used for microarray studies as well in an independent set of 34 additional samples. Integrative analysis of our results with other cervical cancer profiling studies could facilitate the development of multiplex diagnostic markers of cervical cancer progression.

## Introduction

Cervical carcinoma is the second most common cancer affecting women worldwide (age-standardized rate, world, ASR [W], of 15.2), accounting for more than half a million new cases every year [1]. From a study in India, the three most common fatal cancers reported in women at 30–69 years of age were cervical (33,400 [17.1%]), stomach (27,500 [14.1%]), and breast (19,900 [10.2%]) [2]. Persistent infection by high-risk human papillomavirus (HPV) is the central etiological factor for the development of cervical cancer [3]. Although early detection and advancement in diagnostic and treatment modalities have led to improved disease management and increased survival of patients in developed countries, in India cervical carcinoma still continues to be the most common cancer among women and accounts for the maximum cancer deaths each year. Majority of cervical carcinoma are curable if diagnosed at an early stage. Despite this, it causes as many deaths as breast cancer which has an incidence rate nearly double that of cervical cancer [1]. A major reason behind this disparity is the presentation of the disease to the clinics in the late stages. Therefore, biomarkers that could detect the disease early, predict aggressive behavior, and/or define molecular markers for more effective targeted therapy could offer newer insights to improve the existing therapeutic window.

Expression profiling based on microarrays has been used extensively in aiding cancer diagnosis, classifying cancer subtypes, determining prognosis and predicting response to therapy. The first report demonstrating clinically relevant classification of cancer based on microarray was from Golub et al. [4], where a 50-gene signature could distinguish AML (acute myeloid leukemia) and ALL (acute lymphoblastic leukemia) patients with 100% accuracy. Again, using microarray Alizadeh and coworkers identified new subtypes of diffuse large B-cell lymphoma (DLBCL) that correlated with long-term (8–10 year) patient survival [5]. In breast cancer, expression profiling has helped in the identification of ER (estrogen-receptor)-positive and ER-negative cancers as fundamentally distinct diseases at the molecular level [6, 7] as well as shown that prognosis of patients with ER-positive disease is largely determined by the expression of proliferation-related genes [8]. Based on the gene signatures identified from gene expression studies two diagnostic chips have been developed which are now used extensively to take clinical decisions in breast cancer; the FDA-approved MammaPrint assay (Agendia, The Netherlands) and Oncotype DX (Genomic Health, Redwood City, CA) [9, 10]. In prostate cancer, a clinical test based on prostate cancer 3 (*PCA3*) biomarker mRNA in urine, was recently approved in Europe under CE trademark [11].

In cervical cancer, expression profiling studies have helped to identify a number of potential diagnostic markers like mini chromosome maintenance 5 (*MCM5*), cell division cycle protein 6 (*CDC6*) and chromatin licensing and DNA replication factor 1 (*CDT1*) [12, 13]. It has also been used to understand the process of carcinogenesis [14]; to probe for biomarkers of radiation response [15]; and to identify molecular signatures for predicting treatment response [16, 17] and prognosis [18]. But few studies have been done to analyze the molecular changes during the progression of cervical cancer from normal cervix through International Federation of Gynaecology and Obstetrics (FIGO) stages I to IV, based on which treatment decisions are mostly made. This is especially true in the case of Indian cohort of patients where, notwithstanding the overwhelming burden of the disease (1/4th of the global incidence), very few gene expression studies are available [16, 19].

In this study we utilized gene expression profiling to analyze changes in cervical cancer, during the progression from FIGO stage I to III as compared to normal cervix. We also analyzed the expression profiles of cervical cancer samples based on the early or advanced stage of the disease. We have been able to identify a set of genes which could serve as signatures for the progressive stages (FIGO I, II, III) as well as few potential biomarkers that have diagnostic or therapeutic significance. Also by the analysis of expression profiles of cervical cancer at the early and advanced stages we were able to identify potential biomarkers that could serve as therapeutic targets for the advanced stages. We then successfully validated these potential biomarkers in the 29 samples used for microarray as well in an independent set of 34 samples.

## Material and Methods

### Clinical tissue specimens

Frozen cervical tumor biopsies used in the study were obtained through Radiation Oncology Department and Tumour Tissue Repository of the Tata Memorial Hospital, Mumbai, according to the Hospital's Institutional Review Board (IRB) guidelines. Noncancerous cervical tissues were procured from patients who were undergoing hysterectomy for reasons other than cervical cancer, from D. Y. Patil Hospital and Research Centre, Navi Mumbai, after obtaining IRB approval and written informed consent from the patients. The samples were collected in liquid nitrogen and a laboratory code was assigned to maintain confidentiality.

For microarray analysis, cervical cancer samples (*n* = 25) at FIGO stages I (*n* = 8), II (*n* = 9), and III (*n* = 8) and four normal cervix tissues were included. The same samples were used for identifying genes discriminating early (FIGO stage IA, IB, and IIA, *n* = 11) and advanced stages of cervical cancer (FIGO stage IIB, IIIA, and IIIB, *n* = 14). In addition, an independent set of 34 samples comprising normal cervix tissues (*n* = 12) and cervical tumor samples at FIGO stages I (*n* = 3), II (*n* = 9), and III (*n* = 10) were used for validating selected genes.

### Processing of samples and RNA extraction

Tissue samples were first subjected to cryosectioning to collect sections for hematoxylin and eosin (H&E) staining as well as for the isolation of RNA. Microscopic examination of H&E-stained sections of tumor samples was carried out by the pathologist (K. D.) to assess percentage of tumor in the sample, while in nonmalignant cervical samples the stained sections were analyzed to check for any morphologic abnormalities. Normal cervical tissue was represented by normal stratified squamous epithelium and underlying loose connective tissue stroma. Poorly differentiated cervical squamous carcinoma had a sheeted appearance with hardly any stroma visible (Fig. S1). Only those samples with 50% or more tumor cells were considered for microarray studies and validation. Extraction of total RNA from cervical tumor sections was done using RNeasy mini kit (Qiagen, Hilden, Germany), while isolation of RNA using TriZol reagent (Life Technologies, Carlsbad, CA) followed by clean-up using RNeasy mini kit was done for normal cervical tissues. The quality and integrity of the RNA was checked by denaturing agarose gel electrophoresis.

### Expression profiling by microarray

Whole genome mRNA expression profiling of the cervical samples was performed using Human Arrays 19K (University Health Network Microarray Center, Ontario Cancer Institute, Canada). Two-color microarray experiment was carried out using cervical samples as target (labeled with Cy5 fluorescent dye, GE Healthcare, Buckinghamshire, U.K.) and universal human reference RNA, UHRR (Stratagene Corporation, La Jolla, CA) (labeled with Cy3 fluorescent dye, GE Healthcare, Buckinghamshire, U.K.) as reference. Labeling and hybridization were done according to standard protocols. Scanning of the arrays were done on GenePix Professional 4200A Scanner (Axon Instruments, Inc., Molecular Devices, Sunnyvale CA) using the GenePix Pro 5 software. The raw data in .gpr format is available from GEO database (Accession ID GSE46857).

### Microarray data analysis

#### Preprocessing of the data

Analysis of the microarray data was carried out using Agilent Genespring GX11.5 software. The raw data was log2 transformed and normalized using Lowess normalization. For individual analyses, only the probe sets with normalized fluorescence intensity values between 100% and 20% of the whole data, present in at least 75% of the sample cohort were included. Additionally, only the probe sets which were flagged as “Present” or “Marginal” in 100% of the samples in each class were used for further analysis.

### Gene selection, clustering, and pathway analysis

In order to identify genes which were differentially expressed between the normal (N) and different stages of cervical cancer, a supervised method of analysis was adopted. Probe sets with twofold or more difference in average expression in at least one out of six pairs of conditions (N vs. Stage I, N vs. Stage II, N vs. Stage III, Stage II vs. Stage I, Stage III vs. Stage II, and Stage III vs. Stage I) were selected for significance analysis by one-way analysis of variance (ANOVA) at a *P*-value cutoff of 0.05 and using permutative method for *P*-value computation (50,000 permutations). Benjamini and Hochberg false discovery rate (FDR) was used for multiple testing correction [20]. The differentially expressed genes were clustered using Hierarchical clustering analysis based on Pearson correlation and average linkage rule.

In order to define genes differentially expressed between the early and advanced stages of cervical cancer, probe sets with twofold or more difference in average expression between the two conditions were selected. The selected genes were further subjected to unpaired *T*-test with unequal variance (Welch *t*-test) at a *P*-value cutoff of 0.05 using permutative method of *P*-value computation (50,000 permutations).

To identify the biological interactions between the differentially expressed genes and to identify the pathways that differed most between conditions analyzed, the gene sets identified to be differentially expressed were subjected to pathway analysis using KEGG pathway analysis software and Biointerpreter software (Genotypic technology, Bangalore, India).

### Quantitative real-time PCR

Reverse transcription reactions were performed using standard protocols with Superscript™ first strand synthesis kit (Invitrogen, Carlsbad, CA). SYBR green (Invitrogen, Carlsbad, CA) based chemistry was utilized for quantitative real-time polymerase chain reaction (qRT-PCR) reactions. All PCR reactions were performed in duplicate and run on ABI Prism 7900 Sequence Detection System (Life Technologies, Carlsbad, CA). Each sample was normalized based on the average expression of four housekeeping genes (*18S rRNA*, *RPS18*, *TBP*, and *HMBS*). The comparative CT (ΔCT) method was used for quantification of gene expression and relative quantity of the gene of interest was calculated as 2^−ΔCT^. Primer sequences used in the study are listed in Table S1.

Statistical analysis of the real-time data was performed on GraphPad Prism software (GraphPad Software Inc., San Diego, CA). Depending on whether data followed normal distribution or not Welch *t*-test or Mann–Whitney *U* test was used for significance testing. A *P*-value of <0.05 was considered significant. Pearson (linear) correlation analysis between the microarray data and real-time data was also performed. A two-sided *P* ≤ 0.01 and a correlation coefficient of 0.7 were considered significant.

## Results

### Whole genome expression profiling of progressive stages in cervical cancer

Here, expression of 19,200 human genes and expressed sequence tags were compared to identify genes differentially expressed between normal cervix and cervical cancer samples at different progressive FIGO stages (I–III). Significance analysis using one-way ANOVA corrected with Benjamini and Hochberg FDR at *P* ≤ 0.05 and a fold change cutoff of 2 identified 1377 genes to be differentially expressed. Interestingly, overall, there was >80% overlap in the gene panels differentially expressed between normal and successive FIGO stages (1161, 1224, and 1163 genes, respectively, in N vs. Stage I, N vs. Stage II, and N vs. Stage III) (Fig. S2). Thus, though there was evident deregulation of genes in invasive cancer as compared to normal, not much increase in the number of genes deregulated or significant change in the expression of deregulated genes with progression of the disease could be seen (Fig. S3).

In order to identify specific genes deregulated in the initial and later stages of cervical cancer progression, genes differentially expressed compared to normal only in the early stages I and II (*n* = 69) or in late stages II and III (*n* = 56) were examined further. Surprisingly, while individual genes deregulated in the two groups were different, they shared similar functional categories. For instance, both the groups displayed major downregulation of genes involved in cell matrix interaction (*ADAM33*), adhesion (*CALD1*, *CELSR2*), tight junctions (*PARD3B*), and phosphatases (*PPP2R2C*). Additionally, while genes involved in metabolic pathways (*AGA*, *BAAT*, *AGXT2*), RNA splicing (*PRPF4B*) and proteosomal (*PSMD8*) and ribosomal proteins (*RPS6KA1*) were upregulated in Stage I and II, genes related to chromatin remodeling (*PATZ1*), cell division (*DONSON*, *NASP*), and kinases (*SRPK1*) were up in Stage II and III.

### Distinct gene expression signatures defining pathological stages

**Figure 1:**
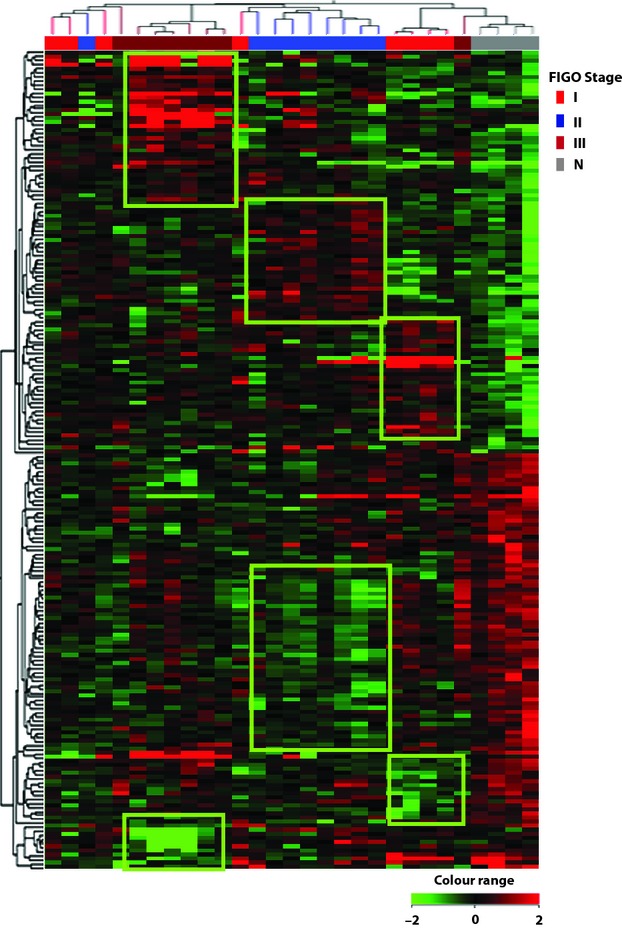
Clustering and TreeView analysis using International Federation of Gynaecology and Obstetrics (FIGO) stage gene signatures. Hierarchical clustering using 201 genes classified the samples based on FIGO stages. The gene clusters in green boxes represents genes over/under-expressed in specific stages.

To identify gene signatures which could distinguish the progressive FIGO stages, genes (*n* = 201) which were deregulated exclusively in Stages I, II, and III (compared to normal) were selected. By using hierarchical clustering based on Pearson correlation and average linkage on these selected genes we were able to delineate the different stages and the normal samples into four definite clusters (Fig. 1). A fifth cluster comprised of a few misclassified samples (three Stage I, one Stage II, and one Stage III). Distinct gene clusters indicating up and downregulated genes in hierarchical cluster were evaluated for their molecular function. These analyses revealed that in Stage I, genes involved in DNA repair and replication (*RPA3*), angiogenesis (*VAV2*), and ERBB signaling (*NRG3*) were upregulated, while genes involved in cytoskeletal remodeling (*DPYSL3*), transcription factor (*TCF4*) and tumor suppressor genes (*SASH1*) were downregulated. Samples in Stage II were marked by upregulation of DNA repair genes (*HYRC*) and genes with functions which may lead to extensive proliferation like the kinases (*NEK2*), growth factors (*PGF*), and genes with mitogenic effect (*FGL1*), while in Stage III, cell cycle regulatory genes (*RCC1*), certain genes related to metastasis (*LAMA5*) and oncogenes (*MECOM*) were overexpressed. Genes highly downregulated in the advanced stages included, tumor suppressors (*NAV3*), phosphatases (*PTPRS*, *DUSP1*), and cytoskeletal proteins (*PKP3*). Thus overall, angiogenesis and proliferation-related genes were overexpressed in the early stages while genes related to metastasis were the ones overexpressed as the cancer progresses.

### Differential expression of genes independent of cancer stages

Comparison of genes differentially expressed in progressive FIGO stages identified 1015 genes common among the three groups, indicating that these genes were deregulated throughout cervical carcinoma progression. Two-way cluster and TreeView analysis based on the expression of these 1015 genes by self-organizing maps (SOM) utilizing Euclidean distance metric could distinctly separate the normal and cancer samples (Fig. S4). Interestingly, comparison of this gene set (668 annotated genes from 1015) with the published literature [14, 21–25] showed considerable overlap (Table S2). The study also identified novel genes of significance; for instance, the DNA repair gene-*BRCA1* expression was found to be upregulated in cervical cancer as compared to normal cervix in this study.

**Figure 2:**
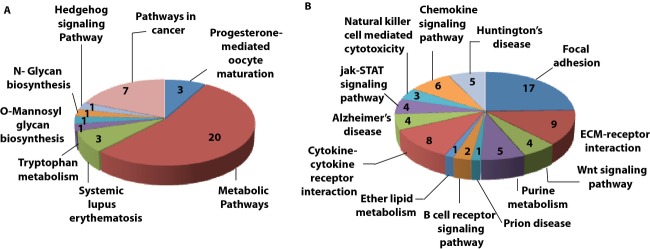
Significantly altered pathways in cervical cancer. The analysis was done using Biointerpreter software. (A) Pathways upregulated. (B) Pathways downregulated.

Altogether, almost 50% of the genes differentially expressed in cervical cancer as compared to normal cervix were upregulated. Pathway analysis of differentially expressed genes showed metabolic pathways, pathways in cancer and signaling pathways to be significantly upregulated, while focal adhesion, cytokine–cytokine receptor interaction, WNT signaling, etc., were significantly downregulated in cancer compared to normal cervix (Fig. 2).

### Genes differentially expressed in advanced versus early stages of cervical cancer

FIGO stages IA to IIA were considered “early,” while progressive stages IIB onward were considered “advanced” as described before [25, 26]. Statistical significance analysis, by unpaired Welch *t*-test using permutative method of *P*-value computation identified 161 genes to be significantly differentially expressed between the groups, at a fold change cutoff of ≥2. Twenty-nine genes were differentially expressed between early and advanced stage of cervical cancer on a stringent analysis at a *P*-value cutoff of 0.01 and fold change cutoff of 2.

As the number of annotated genes in this selected gene list was less, pathway analysis could not identify any of the major pathways as clearly up/downregulated. Analysis of function of the annotated genes in the 161-gene panel, however, indicated that apoptosis-related genes (*BNIP3*, *BCL2L11*, *TNFSF13*, *PERP*, *ATF6*, *BPTF*) and phosphatases (*DUSP1*, *PPP2R5E*, *PTPN4*) were underexpressed whereas genes involved in transcriptional activation (*ATF2*, *GTF3C1*, *TFE3*, *MCRS1*) and signaling, and migration of cells (*MAPRE3*, *PAK7*, *PIK3R1*) were overexpressed in advanced stage as compared to early-stage cervical cancer.

**Figure 3:**
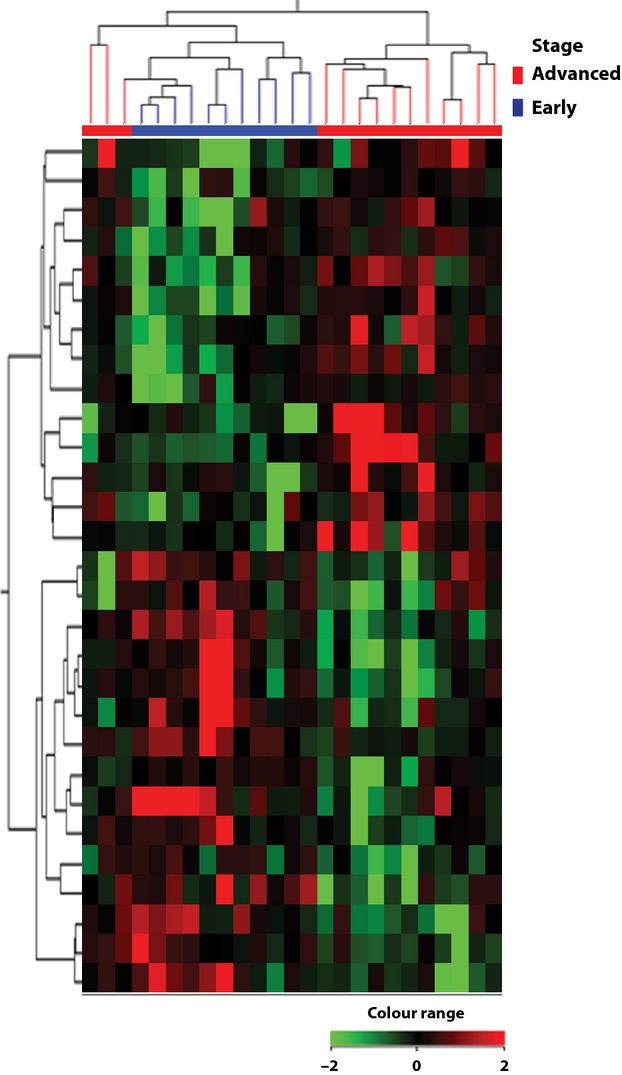
Hierarchical clustering and TreeView analysis of genes differentially expressed in advanced- versus early-stage cervical cancer. Twenty-nine differentially expressed genes at *P* ≤ 0.01 and FC 2 was used for the analysis.

Two-way hierarchical clustering (Pearson correlation and average linkage rule) using the 29 genes (*P* ≤ 0.01 and fold change [FC] = 2) gave two major clusters, one comprising of all of the early-stage samples and second, with most advanced-stage samples (Fig. 3). One of the samples in the advanced stage clustered along with the early-stage samples and two advanced-stage samples clustered separately, and closer to early-stage cluster.

### Real-time RT-PCR (qRT-PCR) evaluation of selected candidate genes in microarray as well as independent samples

**Figure 4:**
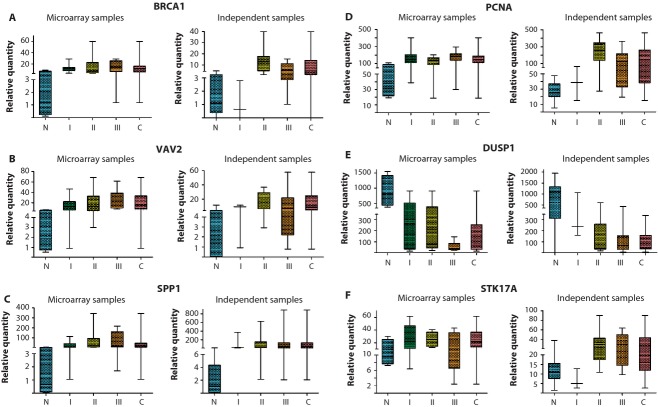
Box plots (A–F) depicting validation of differentially expressed genes by real-time PCR. The difference in expression between Normal and Stages I, II, and III was significant (*P* ≤ 0.05) except in the case of Normal and Stage I in the independent set which had only three samples in the group. N, normal cervix; I, FIGO stage I; II, FIGO stage II; III, FIGO stage III; C, total cervical tumor samples; FIGO, International Federation of Gynaecology and Obstetrics; PCR, polymerase chain reaction.

**Figure 5:**
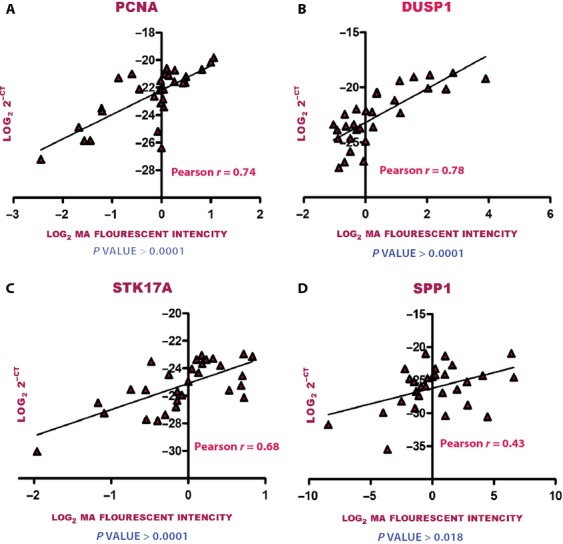
Pearson correlation analysis of microarray and real-time polymerase chain reaction (PCR) data. Microarray fluorescent intensity is given on the *x*-axis and threshold cycle value from real-time PCR data are given on *y*-axis. A: PCNA; B: DUSP1; C: STK17A; D: SPP1.

To confirm differential expression of the genes identified by microarray, a few selected genes were validated by qRT-PCR. From the genes differentially expressed between normal tissue and cervical cancer (1015) we selected nine genes (*BRCA1*, *SPP1*, *PRKAR1B*, *LAMA2*, *PCNA*, *STK17A*, *VAV2*, *DUSP1*, and *APP*), which had either been reported in other cancers or were potential biomarkers as they were secretory proteins. Among these, *LAMA2*, *DUSP1*, *PRKAR1B*, and *APP* were downregulated while the rest of the genes were upregulated in cancer as compared to normal cervical samples in the samples used for microarray. Remarkably, seven out of these nine genes showed significant (*P* ≤ 0.05) difference in expression between the compared groups (Normal vs. cancer, Normal vs. Stage I, Normal vs. Stage II, and Normal vs. Stage III) as determined by microarrays (Fig. 4). To evaluate the degree of correlation between qRT-PCR and microarray data a linear correlation analysis (Pearson) was performed. The analysis revealed significant correlation between the results from the two analysis (*P* ≤ 0.01) (Fig. 5).

Expression of the seven validated genes (*BRCA1*, *SPP1*, *LAMA2*, *PCNA*, *STK17A*, *VAV2*, *DUSP1*) was further analyzed in an independent cohort of 34 cervical samples (12 normal, 3 Stage I, 9 Stage II, and 10 Stage III) by qRT-PCR. Statistically significant differential expression of the genes was observed in this independent sample set as well, further confirming the findings of our microarray analysis (Fig. 4).

**Figure 6:**
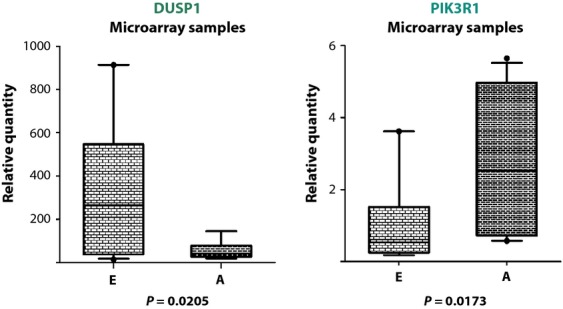
Box plots depicting validation of differentially expressed genes by real-time PCR. DUSP1 was downregulated while PIK3R1 was upregulated in advanced-stage cervical cancer. E, early stage (*n* = 11); A, advanced stage (*n* = 14); PCR, polymerase chain reaction.

From the genes differentially expressed between early and advanced stages of cervical cancer, six genes – three upregulated (*BCL3*, *IGF2*, and *PIK3R1*) and three downregulated (*PTPN4*, *PERP*, and *DUSP1*) were selected for validation by qRT-PCR. Of these, *PIK3R1* and *DUSP1* were found to be significantly differentially expressed in the samples used for microarray (Fig. 6).

## Discussion

Cervical carcinoma is curable in majority of cases if diagnosed early. Routine clinical management of the disease relies on the clinical stage and histological classification as described by FIGO. Though the treatment modality followed is essentially effective in the early stages, with a high 5-year survival rate of >90%, the rate decreases considerably in the later stages to as low as 16.4% in Stage IVA, whereas palliative treatment is the only option available for most advanced Stage IVB and few Stage IVA cancers. Thus, even though histological staging provides valuable information about the clinical behavior of the tumor, it has limited predictive value. Distant metastasis could be observed even in patients with small-volume tumors. Furthermore, the histological appearance of the tumors could not fully reveal the underlying complex genetic alterations and the biologic events involved in their development and progression. Although various clinical predictive and prognostic factors and treatment outcome have been established, molecular events which could predict outcome and divergent behavior need to be investigated further.

In this study, we have attempted to investigate the alterations in the gene expression profile of cervix uteri, as it progresses through various FIGO stages of carcinoma, in a cohort of Indian patients. Expression profiles of cervical cancer samples (*n* = 25) at different progressive FIGO stages (I–III) were compared with normal cervix (*n* = 4). Analysis of the genes differentially expressed between normal cervix and stages of cervical cancer indicated that majority of the genes were deregulated even at the earliest stage of invasive cancer. In fact, there were >80% overlap (1015 of total 1377) in the panel of genes differentially expressed between normal cervix and progressive FIGO stages, indicating that the deregulation of these genes occur early and stays throughout cervical carcinoma progression. An increase in the number of deregulated genes, as well as increase in expression of individual genes with progressive preneoplastic conditions (CIN1, 2, and 3) leading to invasive cancer have been reported earlier [27, 28]. Evaluation of the genes exclusively deregulated either in the initial (Stage I and II) or later stages (Stage II and III) of cervical cancer progression showed that while individual, deregulated genes were different in the two cases, they shared similar functional category. For instance, genes related to cell matrix interaction, adhesion, cytoskeletal reorganization and phosphatases were downregulated while metabolic genes and genes related to cell division and proliferation were upregulated in both the gene panels.

The analysis also revealed few genes differentially expressed uniquely in the different stages. An unsupervised hierarchical clustering using these selected genes (*n* = 201) could separate the different stages and the normal samples into four definite clusters (Fig. 1), though there were a few misclassifications.

Majority of the genes identified to be differentially expressed were altered in cervical cancer as opposed to normal cervix, irrespective of the FIGO stages. A comparison of these genes (668 annotated of 1015) to previous reports showed a considerable overlap (126 genes) with the six published studies (Table S2) [14, 21–25]. Among these, 96 genes were in common with data published by Perez-Plasencia et al. [23] where expression profiles of three normal cervical samples were compared to eight FIGO stage II cervical cancer samples. Wong et al. [25] compared gene expression profiles between 29 cervical cancers and 18 normal cervical tissues and identified 102 genes to be differentially expressed between the comparing groups. Eleven genes reported in their study were in common to the panel of genes identified in this study. Also 11 genes identified in this study were reported to be differentially expressed in three or more of the compared reports. These included some important genes like DNA replication licensing protein *MCM2*, which is tested as biomarker for screening of cervical lesions [29]; *CENPF* which plays a role in chromosome segregation during mitosis and is reported to be frequently amplified in hepatocellular carcinoma [30]; *PRC1* which is involved in cytokinesis [31]; and *CRYAB* a tumor suppressor [32].

Pathway analysis of annotated genes using Biointerpreter software showed metabolic pathways, pathways in cancer and signaling pathways to be significantly upregulated, while focal adhesion, cytokine–cytokine receptor interaction and WNT signaling were significantly downregulated in cervical carcinoma (Fig. 2).

Even as an overlap of genes differentially expressed between normal cervix and cervical cancer was observed among our data and published literature, majority of the genes identified is reported for the first time in cervical cancer. For instance, in the study upregulation of DNA repair gene – *BRCA1* was observed in cancer as compared to normal cervix. Validation of the gene using real-time PCR showed a 9.2-fold increase in *BRCA1* expression in cervical cancer as compared to normal cervix. Over expression of *BRCA1* has been demonstrated in lung cancer and sporadic ovarian cancer where it was reported to lead to chemoresistance [33, 34]. However, to the best of our knowledge increased expression of the gene in cervical cancer has not been reported.

We could also identify other genes of diagnostic or therapeutic significance in the study. One such gene is *SPP1* (secreted phosphoprotein 1 also known as osteopontin [OPN]) that encodes an extracellular glycosylated bone phosphoprotein. Real-time validation study showed the gene to be overexpressed by 34.6-fold in cervical cancer. In a meta-analysis carried out to evaluate OPN as a marker for aggressiveness and patient survival, Weber et al. [35] concluded that OPN was significantly associated with survival in several cancers. OPN has been associated with poor prognosis in head and neck cancer patients [36] and implicated to predict recurrence in oral squamous cell carcinoma [37]. Increased serum concentrations of OPN have been reported in AML, chronic myelogenous leukemia and multiple myeloma [38]. Upregulation of *SPP1* has been reported in expression profiling studies of cervical cancer as well [25, 39]. Clinical significance of elevated OPN in plasma of cervical cancer patients as a diagnostic and prognostic biomarker was evaluated in a study by Cho et al. [40]. Plasma OPN levels were measured in 81 patients with cervical cancer, 34 patients with carcinoma in situ (CIS) of the uterine cervix, and 283 healthy women using enzyme-linked immunosorbent assay (ELISA). As a diagnostic marker for cervical cancer, OPN had a sensitivity and specificity of 50.6% and 95.0%, respectively. SPP1 expression also correlated significantly with overall survival (*P* = 0.002) and disease-free survival (*P* = 0.033) [40].

Another gene of importance validated was *PCNA* (proliferating cell nuclear antigen), which increases the processivity of leading strand synthesis during DNA replication and is involved in the RAD6-dependent DNA repair pathway [41]. Astudillo et al. [42] noted a significant increase in protein expression of *PCNA* with increasing grades of cervical lesions from normal epithelia to invasive squamous cell carcinoma. The E7 oncoprotein of high-risk HPV is known to activate PCNA possibly by abrogation of normal cell cycle control by the E7 oncogene, reverting the p21 (Cip1)-mediated inhibition of PCNA [43], hence the increase in the level of PCNA with progression of the disease. Increased *PCNA* expression was also shown to be related to a shorter disease-free period and overall survival time in patients with breast cancer [44]. The use of *PCNA* along with *Ki-67* has been suggested as specific proliferative markers of prostate cancer for early cancer diagnosis [45].

*DUSP1/MKP1* is a dual specificity phosphatase of MAP kinase pathway that may play an important role in the negative regulation of cellular proliferation. *DUSP1* was downregulated in cervical cancer samples in this study which concurs with published reports [23]. In fact, a progressive reduction in the expression of *DUSP1* was observed as the disease progressed from normal through FIGO stages I–III. Gradual decrease in the expression of the gene has been shown in lung cancer as the tissue type went from normal to increasingly undifferentiated carcinoma [46]. Conversely, a role of *DUSP1* in angiogenesis, invasion and metastasis in non-small-cell lung cancer (NSCLC) cells has also been demonstrated by Moncho-Amor et al. [47] by downregulation of *DUSP1* in H460 cell line. There is a balance between the activation and inactivation of the mitogen-activated protein kinases which is mediated by DUSPs which modulates proliferation or apoptosis in different tissues. In order to understand its role in cervical cancer progression, further studies need to be carried out.

Two genes (*VAV2* and *STK17A*) from the 201-gene panel were selected for validation by qRT-PCR. *VAV2* works as a guanosine nucleotide exchange factor (GEF) that activates different members of the Rho/Rac family of GTPases in a tyrosine phosphorylation-dependent manner [48]. Recent reports provide evidence for its critical role in host-mediated tumor progression and angiogenesis, particularly in tumor endothelium [49]. *STK17A* is a serine/threonine kinase which is known to have apoptosis-inducing activity [50]. It is reported to be downregulated in many cancers and homozygous deletions are noted in melanoma cell line (etoposide-resistant) and laryngeal squamous cell carcinoma cell line [51]. Interestingly overexpression of *STK17A* has been reported in other cancers like glioblastoma where it has been implicated to correlate to poor prognosis in those patients [52]. In this study, *VAV2* was specifically overexpressed in Stage I in the microarray data while *STK17A* was specifically overexpressed in Stage II. However, on real-time validation these genes were found to be overexpressed in other FIGO stages as well.

The 19K microarray data of the three FIGO stages I, II, and III (*n* = 25) were further divided into early stage (IA, IB, IIA) (*n* = 11) and advanced stage (IIB, IIIB) (*n* = 14) with the aim to identify biomarkers and therapeutic targets for the advanced stages. There are only very few studies available comparing early and advanced-stage cervical cancer. In one such study, Wong et al. [25] could identify only two genes *CTGF* and *RGS1* differentially expressed between the groups. Neither of these genes was found to be differentially expressed in our data. *RGS1* was not represented in the 19K chip we used for the study and the difference in expression of *CTGF* between the two groups was not significant in our cohort of patients.

Analysis of function of the annotated genes in the 161-gene panel indicated that apoptosis-related genes (*BNIP3*, *BCL2L11*, *TNFSF13*, *PERP*, *ATF6*, *BPTF*) and phosphatases (*DUSP1*, *PPP2R5E*, *PTPN4*) were underexpressed whereas genes involved in transcriptional activation (*ATF2*, *GTF3C1*, *TFE3*, *MCRS1*) and signaling, and migration of cells (*MAPRE3*, *PAK7*, *PIK3R1*) were overexpressed in advanced stage as compared to early-stage cervical cancer.

Many of the genes which were overexpressed in advanced stages are known to play critical roles in tumor progression, metastasis as well as inducing treatment resistance in many cancers. For instance, *ATF2* (activating transcription factor 2) a basic transcription factor has been reported to be a regulator of radiation and drug resistance in melanomas [53] and known to induce epithelial–mesenchymal transition (EMT) in pancreatic cancer cell lines [54]. Tumor-targeted *ATF2* modulators may be useful as sensitizers in the treatment of *ATF2*-overexpressing tumors [53]. *PIK3R1*, a regulatory subunit of phosphatidylinositol 3-kinase has been implicated in tumorigenesis and metastasis in various cancers. Overexpression of this gene has been associated with advanced stage and poor survival in non-small-cell lung cancer [55]. *PIK3R1* has been suggested as a potential therapeutic target in several cancers including glioblastoma multiforme and NSCLC [55, 56]. Targeting the gene via adeno/lentivirus-mediated shRNA has shown promising results in many preclinical studies [56, 57]. In our microarray data, the gene was overexpressed in advanced stages and was validated by real-time PCR. Another observation was decreased expression of DUSP1 – a phosphatase gene in cervical cancer samples when compared to normal cervix. Underexpression of the gene in advanced stages as compared to early stages could demonstrate a progressive downregulation of this phosphatase with advancement of cervical cancer, suggesting that it may be playing a very critical role in cervical cancer progression. The gene was validated by real-time PCR as well.

Overall, in this study we were able to identify a panel of genes that could distinguish cervical cancer from normal cervix, which included many genes of therapeutic and diagnostic significance. Of particular note is the over expression of the gene SPP1, which could potentially serve as serum diagnostic marker for cervical cancer. Also, *PIK3R1* a gene overexpressed in advanced stages has been suggested as a potential therapeutic target in several cancers. We were able to identify a set of genes that could serve as gene signatures for progressive stages (FIGO I, II, III) of cervical cancer, though studies including larger sample sets need to be undertaken.

## Supporting Information

**Figure S1.** H&E-stained sections of uterine cervical tissue. The panel A represents tissue sections of normal cervix while cervical tumor sections are portrayed in panel B. Normal cervical tissue was represented by normal stratified squamous epithelium and underlying loose connective tissue stroma. Poorly differentiated cervical squamous carcinoma had a sheeted appearance with hardly any stroma visible.

**Figure S2.** Percent overlap between total differentially expressed genes and genes differentially expressed in Stage I, II, and III.

**Figure S3.** Profile plot of expression of 1015 genes across progressive cervical cancer stages as compared to normal. Gene expression did not vary greatly among different cancer stages though there was evident deregulation in transcription as the status changed from normal to cancer. N, normal cervix; I, FIGO stage I; II, FIGO stage II; III, FIGO stage III.

**Figure S4.** Clustering and TreeView analysis of genes differentially expressed in cervical cancer. Clustering using self-organizing map (SOM) of 1015 genes distinctly clustered normal cervical samples together and away from invasive cancer samples.

**Table S1.** List of primers used in the study.

**Table S2.** Overlap of genes differentially expressed between healthy cervical tissue and cervical cancer in the literature.
