# Supplementary material for: Expression profiling of cervical cancers in Indian women at different stages to identify gene signatures during progression of the disease
Source: Cancer Med. 2013 Oct 31;2(6):836–48. doi: 10.1002/cam4.152 (PMC3892388; doi:10.1002/cam4.152)
Supplement: Supplementary file 5 [file cam40002-0836-SD5.docx]

**Table S1: List of primers used in the study**

| **Sl No** | **Gene** | **Primer Sequence (5’-3’)** | **Tm (°C)** | **PCR product size (bp)** |
| --- | --- | --- | --- | --- |
| 1 | RPS18 | **F**-GTGGTGTTGAGGAAAGCAGACA  **R**-TGATCACACGTTCCACCTCATC | 60 | 79 |
| 2 | 18S rRNA | **F**-GTAACCCGTTGAACCCCATT  **R**-CCATCCAATCGGTAGTAGCG | 60 | 151 |
| 3 | APP1 | **F**-ATGCTGGCCTGCTGGCTGAAC  **R**-TGCAGTTCAGGGTAGACTTCTTGGC | 60 | 169 |
| 4 | BCL3 | **F**-TGCACAGAAGCACATGCACCTAC  **R**-GAGGGAGCGAGATGGGGGAACA | 60 | 72 |
| 5 | BRCA1 | **F**-AGCGCCAGTCATTTGCTCCG  **R**-GGACCCAGAGTGGGCAGAGAATG | 60 | 80 |
| 6 | DUSP1 | **F**-GCTGAACTCAGCACATTCGGGAC  **R**-ACACTGAGTCCTTTCTCTTCTGCC | 60 | 87 |
| 7 | HMBS | **F**-GCATCAGCCTGGCCAACTTGTTGC  **R**-ATGGGCATCGTTAAGCTGCCGTGC | 60 | 80 |
| 8 | IGF2 | **F**-GGAGGATTGACAGCAGACTTGCAG  **R**-GAGCGCATAAAGCTAAGGAGGGGT | 60 | 156 |
| 9 | LAMA2 | **F**-TGAGGTGTACCTGCACCCATCTG  **R**-ACGGACTGGAAAATGTGTGCCAT | 60 | 94 |
| 10 | PCNA | **F**-GTCTGCAGATGTACCCCTTGTTGTAG  **R**-TCCTCGATCTTGGGAGCCAAGTAG | 60 | 84 |
| 11 | PERP | **F**-TCATGGTCCAAACCTGTTGCCATAG  **R**-AGCATTTTCCCACACCCTAACCCT | 60 | 113 |
| 12 | PIK3R1 | **F**-CTGCCTAAGAACAGAGTGTGAAGGC  **R**-ACTCCCCCTTCCCAAAGCTAACAT | 60 | 116 |
| 13 | PRKAR1B | **F**-CCTTCCCCGGACTCACTTTTTGGA  **R**-GATGCATTTTGTCCGCTTGTCCT | 60 | 79 |
| 14 | PTPN4 | **F**-CCGTTTGGACAGACCACTTCCAC  **R**-CTTCAGTTCTCCCACAGTACCGGA | 64 | 92 |
| 15 | SPP1 | **F**-TTCTGGAAGTTCTGAGGAAAAGCAGC  **R**-CTGAGATGGGTCAGGGTTTAGCCA | 60 | 82 |
| 16 | STK17A | **F**-TGATGTTTTGTCTGAGTCGGCTGTTG  **R**-TCTTCAGCAGTGGCTCGATCTTCAGG | 60 | 81 |
| 17 | TBP | **F**-GGACTGACCCCACAGCCTATTCAG  **R**-TGTTGTTGCTGCTGCTGCCTTTG | 60 | 80 |
| 18 | VAV2 | **F**-TACCCCTGGTTTGCAGGTAAC  **R**-GCTTATTGCAAAGCGCTCA | 60 | 120 |

**Table S2: Overlap of genes differentially expressed between healthy cervical tissue and cervical cancer in the literature**

| **Sl No** | **Gene Name** | **1015 gene list** | **(Chen et al., 2003)** | **(Ahn et al., 2004)** | **(Santin et al., 2005)** | **(Wong et al., 2006)** | **(Perez-Plasencia et al., 2007)** | **(Biewenga et al., 2008)** |
| --- | --- | --- | --- | --- | --- | --- | --- | --- |
| 1 | **AKAP13** | **X** |  |  |  |  | **X** |  |
| 2 | **ABHD3** | **X** |  |  |  |  | **X** |  |
| 3 | **ACTA2** | **X** |  |  |  |  | **X** |  |
| 4 | **ACADVL** | **X** |  |  |  |  | **X** |  |
| 5 | **AKR1C3** | **X** |  |  |  |  | **X** |  |
| 6 | **ALPP** | **X** |  |  |  |  | **X** |  |
| 7 | **APP** | **X** |  |  |  |  | **X** |  |
| 8 | **ANGPTL2** | **X** |  |  |  |  | **X** |  |
| 9 | **ANXA3** | **X** |  |  | **X** |  |  |  |
| 10 | **APOB** | **X** |  |  |  |  | **X** |  |
| 11 | **APOD** | **X** |  |  |  |  | **X** |  |
| 12 | **ASPN** | **X** |  |  |  |  | **X** |  |
| 13 | **BBS4** | **X** |  |  |  |  | **X** |  |
| 14 | **BUB1B** | **X** |  |  |  |  |  | **X** |
| 15 | **CALD1** | **X** |  |  |  |  | **X** |  |
| 16 | **CNN1** | **X** |  |  |  |  | **X** |  |
| 17 | **CFLAR** | **X** |  |  | **X** |  |  |  |
| 18 | **CAV2** | **X** |  |  |  |  | **X** |  |
| 19 | **CKS1B** | **X** |  |  | **X** |  |  |  |
| 20 | **CXCL12** | **X** |  |  |  |  | **X** |  |
| 21 | **CXCL14** | **X** |  |  |  |  | **X** |  |
| 22 | **CHRD** | **X** |  |  |  |  | **X** |  |
| 23 | **COL3A1** | **X** |  |  |  |  | **X** |  |
| 24 | **COL6A1** | **X** |  |  |  |  | **X** |  |
| 25 | **COL17A1** | **X** |  |  | **X** |  |  |  |
| 26 | **CNTNAP2** | **X** |  |  |  |  | **X** |  |
| 27 | **CCNA1** | **X** |  |  | **X** |  |  |  |
| 28 | **CYYR1** | **X** |  |  |  |  | **X** |  |
| 29 | **DPF3** | **X** |  |  |  |  | **X** |  |
| 30 | **DCN** | **X** |  |  |  |  | **X** |  |
| 31 | **DPT** | **X** |  |  |  |  | **X** |  |
| 32 | **D4S234E** | **X** |  |  | **X** |  |  |  |
| 33 | **DUSP1** | **X** |  |  |  |  | **X** |  |
| 34 | **DUSP8** | **X** |  |  |  |  | **X** |  |
| 35 | **GRIA4** | **X** |  |  |  |  | **X** |  |
| 36 | **GLRX** | **X** |  |  | **X** |  |  |  |
| 37 | **GPX3** | **X** |  |  |  | **X** |  |  |
| 38 | **GPD2** | **X** |  |  |  |  |  | **X** |
| 39 | **IGHG1** | **X** |  |  |  |  | **X** |  |
| 40 | **IGL@** | **X** |  | **X** |  |  |  |  |
| 41 | **IGF1** | **X** |  |  |  |  | **X** |  |
| 42 | **IGFBP4** | **X** |  |  | **X** |  |  |  |
| 43 | **IGFBP5** | **X** |  |  |  |  | **X** |  |
| 44 | **IGFBP6** | **X** |  |  |  |  | **X** |  |
| 45 | **IL1R2** | **X** |  |  | **X** |  |  |  |
| 46 | **IL16** | **X** |  |  |  |  | **X** |  |
| 47 | **JUN** | **X** |  |  |  |  | **X** |  |
| 48 | **KRT1** | **X** |  |  |  | **X** |  |  |
| 49 | **KRT10** | **X** |  |  |  | **X** |  |  |
| 50 | **LAMA2** | **X** |  |  |  |  | **X** |  |
| 51 | **LAMP2** | **X** |  |  |  |  | **X** |  |
| 52 | **MMP2** | **X** |  |  |  |  | **X** |  |
| 53 | **MEMO1** | **X** | **X** |  |  |  |  |  |
| 54 | **MEIS1** | **X** |  |  |  |  | **X** |  |
| 55 | **MSRB3** | **X** |  |  |  |  | **X** |  |
| 56 | **METAP2** | **X** |  |  |  |  | **X** |  |
| 57 | **MCEE** | **X** |  |  |  |  | **X** |  |
| 58 | **MCM6** | **X** |  |  |  |  |  | **X** |
| 59 | **MYO1B** | **X** |  |  | **X** |  |  |  |
| 60 | **MYH11** | **X** |  |  |  |  | **X** |  |
| 61 | **MYL7** | **X** |  |  |  |  | **X** |  |
| 62 | **NRD1** | **X** |  |  |  |  | **X** |  |
| 63 | **NFATC2IP** | **X** |  |  |  |  | **X** |  |
| 64 | **NCOA1** | **X** |  |  |  |  | **X** |  |
| 65 | **NR2F2** | **X** |  |  |  |  | **X** |  |
| 66 | **OSR2** | **X** |  |  |  |  | **X** |  |
| 67 | **OLFML3** | **X** |  |  |  |  | **X** |  |
| 68 | **PRRX1** | **X** |  |  |  |  | **X** |  |
| 69 | **PELI1** | **X** |  |  | **X** |  |  |  |
| 70 | **PI16** | **X** |  |  |  |  |  | **X** |
| 71 | **PER1** | **X** |  |  |  |  | **X** |  |
| 72 | **PMP22** | **X** |  |  |  |  | **X** |  |
| 73 | **PPAP2B** | **X** |  |  |  |  | **X** |  |
| 74 | **PGM5** | **X** |  |  |  |  | **X** |  |
| 75 | **PIK3R1** | **X** |  |  |  |  | **X** |  |
| 76 | **PLN** | **X** |  |  |  |  | **X** |  |
| 77 | **PECAM1** | **X** |  |  |  |  | **X** |  |
| 78 | **PDGFRA** | **X** |  |  |  |  | **X** |  |
| 79 | **PDGFRB** | **X** |  |  |  |  | **X** |  |
| 80 | **PTRF** | **X** |  |  |  |  | **X** |  |
| 81 | **PRICKLE1** | **X** |  |  |  |  | **X** |  |
| 82 | **PRCP** | **X** |  |  |  |  | **X** |  |
| 83 | **PTGIS** | **X** |  |  |  |  | **X** |  |
| 84 | **PPP2R5C** | **X** |  |  |  |  | **X** |  |
| 85 | **RAPGEF3** | **X** |  |  |  |  | **X** |  |
| 86 | **RAMP1** | **X** |  |  |  |  | **X** |  |
| 87 | **RGS5** | **X** |  |  |  |  | **X** |  |
| 88 | **ARHGAP10** | **X** |  |  |  |  | **X** |  |
| 89 | **RYR2** | **X** |  |  |  |  | **X** |  |
| 90 | **S100A4** | **X** |  |  |  |  | **X** |  |
| 91 | **SPP1** | **X** |  |  |  | **X** |  |  |
| 92 | **SDPR** | **X** |  |  |  |  | **X** |  |
| 93 | **SNAI2** | **X** |  |  |  |  | **X** |  |
| 94 | **SNX24** | **X** |  |  |  |  | **X** |  |
| 95 | **SPARCL1** | **X** |  |  |  |  | **X** |  |
| 96 | **SOCS2** | **X** |  |  |  |  | **X** |  |
| 97 | **SYN1** | **X** |  |  |  |  | **X** |  |
| 98 | **SYNPO2** | **X** |  |  |  |  | **X** |  |
| 99 | **TBC1D1** | **X** |  | **X** |  |  |  |  |
| 100 | **TNXB** | **X** |  |  |  |  | **X** |  |
| 101 | **THBS1** | **X** |  |  |  |  | **X** |  |
| 102 | **TRIP13** | **X** |  |  | **X** |  |  |  |
| 103 | **TFPI2** | **X** |  |  | **X** |  |  |  |
| 104 | **TCEAL4** | **X** |  |  |  |  | **X** |  |
| 105 | **TLE1** | **X** |  |  |  |  | **X** |  |
| 106 | **TFRC** | **X** |  |  |  | **X** |  |  |
| 107 | **TFR2** | **X** |  |  |  |  | **X** |  |
| 108 | **TGFBR3** | **X** |  |  |  |  | **X** |  |
| 109 | **TGM3** | **X** |  |  |  | **X** |  |  |
| 110 | **UBR1** | **X** |  |  |  |  | **X** |  |
| 111 | **UXS1** | **X** |  |  |  |  | **X** |  |
| 112 | **VGLL1** | **X** |  |  |  |  | **X** |  |
| 113 | **VIM** | **X** |  |  |  |  | **X** |  |
| 114 | **ZFHX4** | **X** |  |  |  |  | **X** |  |
| 115 | **ZFP36** | **X** |  |  |  |  | **X** |  |
| 116 | **C1QTNF7** |  |  |  |  |  | **X** | **X** |
| 117 | **COL1A1** |  | **X** | **X** |  |  |  |  |
| 118 | **COL1A2** |  | **X** |  |  |  | **X** |  |
| 119 | **LAMC2** |  | **X** |  | **X** |  |  |  |
| 120 | **MGP** |  | **X** |  |  |  | **X** |  |
| 121 | **MCM5** |  |  |  | **X** | **X** |  | **X** |
| 122 | **SPA17** |  |  | **X** |  |  | **X** |  |
| 123 | **TK1** |  | **X** |  |  |  |  | **X** |
| 124 | **TMPRSS4** |  | **X** |  | **X** |  |  |  |
| 125 | **MYBL2** |  | **X** |  | **X** |  |  |  |
| 126 | **ADPRT** |  |  |  | **X** |  |  | **X** |
| 127 | **A2M** |  |  | **X** |  |  | **X** |  |
| 128 | **APLP2** |  |  | **X** |  |  | **X** |  |
| 129 | **APOL2** |  |  | **X** |  |  | **X** |  |
| 130 | **APOL1** |  | **X** |  | **X** |  |  |  |
| 131 | **BAG2** |  |  |  | **X** |  | **X** |  |
| 132 | **BST2** |  | **X** | **X** |  |  |  |  |
| 133 | **BRD3** |  |  |  | **X** |  | **X** |  |
| 134 | **BUB1** |  |  |  |  |  | **X** | **X** |
| 135 | **CDH3** |  |  |  | **X** | **X** |  |  |
| 136 | **CACNB2** |  |  |  |  |  | **X** | **X** |
| 137 | **CTSB** |  |  |  | **X** |  | **X** |  |
| 138 | **CDC6** |  |  |  | **X** |  |  | **X** |
| 139 | **CDC25C** |  |  |  | **X** |  | **X** |  |
| 140 | **CENPA** |  |  |  | **X** |  | **X** |  |
| 141 | **C20orf1** |  |  |  | **X** |  |  | **X** |
| 142 | **CKB** |  |  |  | **X** |  | **X** |  |
| 143 | **CRYL1** |  |  |  | **X** |  | **X** |  |
| 144 | **CCNA2** |  |  |  | **X** |  |  |  |
| 145 | **CCNB1** |  |  |  | **X** |  |  | **X** |
| 146 | **CCNB2** |  |  |  | **X** |  |  | **X** |
| 147 | **CCND2** |  |  |  | **X** |  | **X** |  |
| 148 | **CCNF** |  |  |  | **X** |  | **X** |  |
| 149 | **CSTA** |  |  |  | **X** |  | **X** |  |
| 150 | **CDT1** |  |  |  | **X** |  |  | **X** |
| 151 | **DONSON** |  |  |  | **X** |  |  | **X** |
| 152 | **FOLR3** |  |  |  | **X** |  | **X** |  |
| 153 | **GTSE1** |  |  |  | **X** |  |  | **X** |
| 154 | **C4.4A** |  |  |  | **X** |  | **X** |  |
| 155 | **HSPCA** |  |  | **X** |  |  | **X** |  |
| 156 | **HDLBP** |  |  | **X** |  |  | **X** |  |
| 157 | **H2AV** |  |  |  | **X** |  |  | **X** |
| 158 | **ABCG2** |  |  |  |  |  | **X** | **X** |
| 159 | **BLM** |  |  |  |  |  | **X** | **X** |
| 160 | **CSN1S1** |  |  |  |  |  | **X** | **X** |
| 161 | **CD34** |  |  |  |  |  | **X** | **X** |
| 162 | **CDCA1** |  |  |  |  |  | **X** | **X** |
| 163 | **NUDT10** |  |  |  |  |  | **X** | **X** |
| 164 | **DAAM2** |  |  |  |  |  | **X** | **X** |
| 165 | **EZH2** |  |  |  |  |  | **X** | **X** |
| 166 | **EFNA1** |  |  |  |  | **X** |  | **X** |
| 167 | **ECT2** |  |  |  |  | **X** |  | **X** |
| 168 | **FANCA** |  |  |  |  |  | **X** | **X** |
| 169 | **GPR124** |  |  |  |  |  | **X** | **X** |
| 170 | **HSPB2** |  |  |  |  |  | **X** | **X** |
| 171 | **KIF2C** |  |  |  |  |  | **X** | **X** |
| 172 | **KIF4A** |  |  |  |  |  | **X** | **X** |
| 173 | **LGI4** |  |  |  |  |  | **X** | **X** |
| 174 | **NETO2** |  |  |  |  |  | **X** | **X** |
| 175 | **SLC2A13** |  |  |  |  |  | **X** | **X** |
| 176 | **TENC1** |  |  |  |  |  | **X** | **X** |
| 177 | **TU3A** |  |  |  |  |  | **X** | **X** |
| 178 | **HIP1** |  |  |  | **X** |  | **X** |  |
| 179 | **FLJ10156** |  |  |  | **X** |  |  | **X** |
| 180 | **FLJ20311** |  |  |  | **X** |  |  | **X** |
| 181 | **FLJ21511** |  |  |  | **X** | **X** |  |  |
| 182 | **HIF3A** |  |  |  |  |  | **X** | **X** |
| 183 | **ITGA3** |  | **X** | **X** |  |  |  |  |
| 184 | **IL1RN** |  |  |  | **X** | **X** | **X** |  |
| 185 | **KLK7** |  |  |  | **X** | **X** |  |  |
| 186 | **LMO2** |  |  |  | **X** |  | **X** |  |
| 187 | **HLA-C** |  |  | **X** |  |  | **X** |  |
| 188 | **HLA-DQB1** |  |  | **X** | **X** |  |  |  |
| 189 | **HLA-DRB5** |  |  | **X** |  |  | **X** |  |
| 190 | **MRC2** |  |  |  | **X** |  | **X** |  |
| 191 | **MPPE1** |  |  |  | **X** |  | **X** |  |
| 192 | **NDRG2** |  |  |  | **X** |  | **X** |  |
| 193 | **NDRG4** |  |  |  | **X** | **X** |  |  |
| 194 | **NDN** |  |  |  | **X** |  | **X** |  |
| 195 | **NRP2** |  |  |  | **X** |  | **X** |  |
| 196 | **NEK2** |  |  |  | **X** |  | **X** |  |
| 197 | **ANKT** |  |  |  | **X** |  |  | **X** |
| 198 | **OIP5** |  |  |  | **X** |  |  | **X** |
| 199 | **OLR1** |  |  |  | **X** |  | **X** |  |
| 200 | **PAX8** |  |  |  | **X** |  | **X** |  |
| 201 | **PTHLH** |  | **X** |  | **X** |  |  |  |
| 202 | **PPARD** |  |  |  | **X** |  | **X** |  |
| 203 | **PCNP** |  |  | **X** |  |  | **X** |  |
| 204 | **GART** |  |  |  | **X** |  |  | **X** |
| 205 | **PTTG1** |  |  |  | **X** |  |  | **X** |
| 206 | **PLK** |  |  |  | **X** |  |  | **X** |
| 207 | **POLQ** |  |  |  | **X** |  | **X** |  |
| 208 | **PP1201** |  |  |  | **X** |  | **X** |  |
| 209 | **PTPRZ1** |  |  |  | **X** |  | **X** |  |
| 210 | **RRAS** |  |  |  | **X** |  | **X** |  |
| 211 | **RBPMS** |  |  |  | **X** |  | **X** |  |
| 212 | **S100A9** |  |  |  | **X** |  | **X** |  |
| 213 | **SALL1** |  |  |  | **X** |  | **X** |  |
| 214 | **SEPP1** |  |  | **X** |  |  | **X** |  |
| 215 | **SERPINF1** |  |  |  | **X** |  | **X** |  |
| 216 | **SERPING1** |  |  |  | **X** |  | **X** |  |
| 217 | **SPRR1B** |  |  |  | **X** |  | **X** |  |
| 218 | **SPRR2A** |  |  | **X** |  |  | **X** |  |
| 219 | **SPRR2B** |  |  |  | **X** | **X** |  |  |
| 220 | **TNC** |  |  |  | **X** |  | **X** |  |
| 221 | **TIMELESS** |  |  |  | **X** |  |  | **X** |
| 222 | **TROAP** |  |  |  | **X** |  |  | **X** |
| 223 | **TNFRSF6B** |  |  |  | **X** |  | **X** |  |
| 224 | **UBE2C** |  |  |  | **X** |  |  | **X** |
| 225 | **BM039** |  |  |  | **X** |  |  | **X** |
| 226 | **UPK1B** |  |  |  | **X** |  | **X** |  |
| 227 | **MAF** |  |  |  | **X** |  | **X** |  |
| 228 | **WHSC1** |  |  |  | **X** |  | **X** |  |
| 229 | **ZWINT** |  |  |  | **X** |  |  | **X** |
| 230 | **BBOX1** |  |  |  |  | **X** | **X** |  |
| 231 | **CRISP3** |  |  |  |  | **X** | **X** |  |
| 232 | **DESC1** |  |  |  |  | **X** | **X** |  |
| 233 | **E2F7** |  |  |  |  |  | **X** | **X** |
| 234 | **EDN3** |  |  |  |  | **X** | **X** |  |
| 235 | **HOXA2** |  |  |  |  |  | **X** |  |
| 236 | **HPGD** |  |  |  |  | **X** | **X** |  |
| 237 | **IL18** |  |  |  |  | **X** | **X** |  |
| 238 | **IVL** |  |  |  |  | **X** | **X** |  |
| 239 | **KLK13** |  |  |  |  | **X** | **X** |  |
| 240 | **KRT4** |  |  |  |  | **X** | **X** |  |
| 241 | **KRT7** |  |  |  |  | **X** | **X** |  |
| 242 | **LGR5** |  |  |  |  | **X** | **X** |  |
| 243 | **MLLT4** |  |  |  |  | **X** | **X** |  |
| 244 | **PDCD4** |  |  |  |  | **X** | **X** |  |
| 245 | **PLSCR1** |  |  |  |  | **X** | **X** |  |
| 246 | **PPL** |  |  |  |  | **X** | **X** |  |
| 247 | **PPP1R3C** |  |  |  |  | **X** | **X** |  |
| 248 | **SLURP1** |  |  |  |  | **X** | **X** |  |
| 249 | **SPINK5** |  |  |  |  | **X** | **X** |  |
| 250 | **SPRR2C** |  |  |  |  | **X** | **X** |  |
| 251 | **SULT2B1** |  |  |  |  | **X** | **X** |  |
| 252 | **AEBP1** | **X** |  | **X** |  |  | **X** |  |
| 253 | **CDH13** | **X** |  |  | **X** |  | **X** |  |
| 254 | **CENPF** | **X** |  |  | **X** |  |  | **X** |
| 255 | **CRYAB** | **X** |  |  |  | **X** | **X** |  |
| 256 | **DSG1** | **X** |  |  |  | **X** | **X** |  |
| 257 | **EDNRA** | **X** |  |  | **X** |  | **X** |  |
| 258 | **JAM2** | **X** |  |  |  |  | **X** | **X** |
| 259 | **KRT13** | **X** |  |  |  | **X** | **X** |  |
| 260 | **MAL** | **X** |  |  |  | **X** | **X** |  |
| 261 | **PRC1** | **X** |  |  | **X** |  |  | **X** |
| 262 | **CRIP1** |  | **X** |  | **X** |  | **X** |  |
| 263 | **PLAU** |  | **X** |  | **X** |  |  | **X** |
| 264 | **CDKN2A** |  |  |  | **X** | **X** |  | **X** |
| 265 | **GLTP** |  |  |  | **X** | **X** | **X** |  |
| 266 | **ALOX12** |  |  |  |  | **X** | **X** | **X** |
| 267 | **MCM4** |  | **X** |  | **X** |  |  | **X** |
| 268 | **RHCG** |  |  |  | **X** | **X** | **X** |  |
| 269 | **SPRR1A** |  |  |  | **X** | **X** | **X** |  |
| 270 | **SPRR3** |  |  |  | **X** | **X** | **X** |  |
| 271 | **SPAG5** |  |  |  | **X** |  | **X** | **X** |
| 272 | **TYMS** |  |  |  | **X** | **X** |  | **X** |
| 273 | **MCM2** | **X** |  |  | **X** | **X** |  | **X** |
| 274 | **TOP2A** |  | **X** | **X** | **X** |  |  | **X** |

**X:** up-regulated in cervical cancer in the study, **X:** down-regulated in cervical cancer in the study
